# An OpenCL-Based FPGA Accelerator for Faster R-CNN

**DOI:** 10.3390/e24101346

**Published:** 2022-09-23

**Authors:** Jianjing An, Dezheng Zhang, Ke Xu, Dong Wang

**Affiliations:** 1 Institute of Information Science, Beijing Jiaotong University, Beijing 100044, China; 2 Beijing Key Laboratory of Advanced Information Science and Network Technology, Beijing Jiaotong University, Beijing 100044, China

**Keywords:** convolutional neural network, Faster R-CNN, FPGA, hardware accelerator

## Abstract

In recent years, convolutional neural network (CNN)-based object detection algorithms have made breakthroughs, and much of the research corresponds to hardware accelerator designs. Although many previous works have proposed efficient FPGA designs for one-stage detectors such as Yolo, there are still few accelerator designs for faster regions with CNN features (Faster R-CNN) algorithms. Moreover, CNN’s inherently high computational complexity and high memory complexity bring challenges to the design of efficient accelerators. This paper proposes a software-hardware co-design scheme based on OpenCL to implement a Faster R-CNN object detection algorithm on FPGA. First, we design an efficient, deep pipelined FPGA hardware accelerator that can implement Faster R-CNN algorithms for different backbone networks. Then, an optimized hardware-aware software algorithm was proposed, including fixed-point quantization, layer fusion, and a multi-batch Regions of interest (RoIs) detector. Finally, we present an end-to-end design space exploration scheme to comprehensively evaluate the performance and resource utilization of the proposed accelerator. Experimental results show that the proposed design achieves a peak throughput of 846.9 GOP/s at the working frequency of 172 MHz. Compared with the state-of-the-art Faster R-CNN accelerator and the one-stage YOLO accelerator, our method achieves 10× and 2.1× inference throughput improvements, respectively.

## 1. Introduction

CNN has made significant breakthroughs in many application scenarios in computer vision, such as image classifications [1,2,3], object detection [4,5,6,7], speech recognition [8], etc. In recent years, FPGA-based object detection accelerators have made great progress in real-world applications, such as autonomous driving, smart security systems, etc.

It is a huge challenge to deploy a CNN-based object detection network model that is computationally intensive and storage intensive to mobile devices with limited resources (such as smartphones, smart wearable devices, etc.). As shown in Table 1, the Faster R-CNN detection model [4], whose backbone network is vgg16 [2], requires up to 271.7 billion floating point operations (FLOPS) and more than 137 Megabytes(MB) of model parameters. Therefore, we need to choose a suitable computing platform for object detection. Recent studies have shown [9] that the computing capacity of a typical CPU can only reach 10–100 Giga Floating-point Operations Per Second (GFLOPS), and the energy consumption efficiency is normally below 1 Giga Operation Per Joule (GOP/J). In contrast, the computing power of GPU can be as high as 10 Tera Operation Per Second (TOP/s), which is a good choice for object detection applications. However, GPUs can usually only conduct 32-bit or 16-bit floating point operations and heavily rely on off-chip storage, which makes power consumption high (typical GPUs exceed 200 W). In addition, FPGAs are becoming a candidate platform for energy-saving and low-latency neural network acceleration processing through hardware design for neural networks. FPGA can perform data-parallel and task-parallel computing simultaneously to help improve efficiency. The flexibility of FPGA can also leave more room for the realization and optimization of neural network algorithm functions. Furthermore, FPGA-based CNN hardware accelerator designs [10,11,12,13,14,15] are rapidly developing due to their reconfigurability and fast development time, especially when FPGA vendors provide high-level synthesis (HLS) tools. In [10] proposed a design space exploration method by optimizing the computing resources and external memory access of the CNN accelerator, but they only implemented the convolutional layer. The author of [13] proposed a fixed-point CNN accelerator design scheme based on the OpenCL framework. However, because their convolution implementation method was based on the matrix multiplication mode and the device core separation design, the advantages of FPGA’s deep pipeline characteristics were not been tapped to achieve a higher computing efficiency and smaller storage bandwidth.

Due to the higher computational complexity of object detection algorithms and their more complex network designs, hardware accelerator designs [16,17,18,19,20,21,22,23] for CNN-based object detection algorithms are still rare from both the computing and storage perspectives. Table 1 shows that the two-stage Faster R-CNN detection algorithm is more computationally expensive than the single-stage Yolo detection algorithm by 5×–7×, so almost all FPGA-based object detection accelerator designs only consider single-stage detection algorithms, such as Yolo [18], Yolov2 [17,19,24], Yolov3 [22,23], etc. Due to the two-stage detection algorithm, Faster R-CNN usually has an improved recognition accuracy for small objects compared to the one-stage detection algorithm [4]. The optimization flow proposed in work [16] can implement a Faster R-CNN; however, the peak performance and bandwidth utilization of the design are greatly limited due to its use of a 32-bit floating-point format. The work of [18] presents a high-performance hardware implementation of Faster R-CNN and Yolov1 [6] on FPGA. However, their work only implements convolution computations on FPGA and fully connected layer computations on CPU. This design is very unfriendly to resource-constrained embedded platforms, because the CPUs of embedded platforms are generally limited in terms of their computing power.

The reason for the large amount of parameters and calculation of the Faster R-CNN detection algorithm is that it includes a fully connected(fc) layer with a large amount of parameters and region proposals. Each region proposal needs to complete the calculation of the fully connected layer just like a complete picture, and this will bring great obstacles to memory and bandwidth, especially when applied to embedded FPGA platforms. In this paper, we have studied how to deploy a complete Faster R-CNN object detection accelerator on FPGA platform. An efficient and scalable hardware accelerator design for Faster R-CNN object detection based on OpenCL is proposed. Specifically, this paper makes the following contributions:

- We propose an OpenCL-based deep pipelined object detection hardware accelerator design, which can implement Faster R-CNN algorithms for different backbone networks (such as vgg16 [2], resnet50 [3]). To our knowledge, we are the first to systematically analyze and design a Faster R-CNN object detection accelerator.
- We perform hardware-aware algorithm optimizations on the Faster R-CNN network, including quantization, layer fusion, and a multi-batch RoIs detector. The cost of quantizing the network is a less than 1% accuracy loss and the multi-batch RoIs detector method can help the network to increases its speed by up to 11.1×. This greatly improves the utilization of hardware resources and bandwidth, maximizing the performance gains of the final design.
- We introduce an end-to-end design space exploration flow for the proposed accelerator, which can comprehensively evaluate the performance and hardware resource utilization of the accelerator to fully exploit the potential of the accelerator.
- Experimental results show that the proposed accelerator design achieves a peak throughput of 846.9 GOP/s at a working frequency of 172 MHz. Compared with the state-of-the-art Faster R-CNN accelerator and the one-stage YOLO accelerator, our method achieves 10× and 2.1× inference throughput improvements, respectively.

## 2. Preliminaries

This section mainly reviews the Faster R-CNN [4] object detection algorithm and OpenCL-based heterorgeneous computing platform setup.

### 2.1. Review of the Faster R-CNN Algorithm

After the development of R-CNN [7] and Fast R-CNN [5], Faster R-CNN [4] is the most classic object detection algorithm in the two-stage object detection algorithm to date. Faster R-CNN was created to solve the bottleneck of candidate region extraction and further share the convolution operation. Faster R-CNN is the first object detection algorithm to achieve end-to-end training.

**Figure 1:**
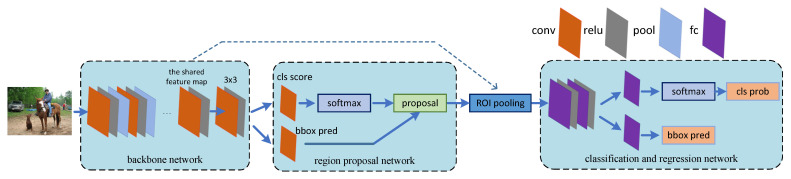
Faster R-CNN object detection algorithm flow.

More specifically, Figure 1 shows the entire Faster R-CNN object detection algorithm flow. Faster R-CNN mainly consists of four essential parts: backbone network, region proposal network (RPN), region of interest (RoI) pooling layer, and classification and regression network. The first part is the backbone network, which includes the preprocessing of the input image and the forward computation of CNN. Generally speaking, this consists of some typical CNN networks, such as vgg16 [2] and resnet50 [3], which are mainly used to extract the features of the pictures. The last convolutional layer of the backbone network is used as a shared convolutional layer, and the output feature map is used as the input of RPN and RoI pooling.

RPN is the second part of Faster R-CNN, used to generate region proposals, which are the regions of interest in the network. Classical detection methods are very time-consuming when generating region proposal. For example, R-CNN [7] uses the Selective Search(SS) [26] method to generate region proposals. Faster R-CNN uses RPN to generate regional proposals, abandoning the traditional sliding window and SS methods, which significantly improves the generation speed of region proposals. Specifically, from Figure 1, we can see that the RPN is divided into two routes. The upper route is used to classify anchors in softmax to obtain the foreground and background (the detection target is foreground) and the lower route is used to calculate the bounding box regression offset for the anchors to obtain accurate region proposals. The final proposal layer is responsible for integrating foreground anchors and bounding box regression offsets to obtain proposals, and eliminate proposals that are too small and beyond the boundary.

**Figure 2:**
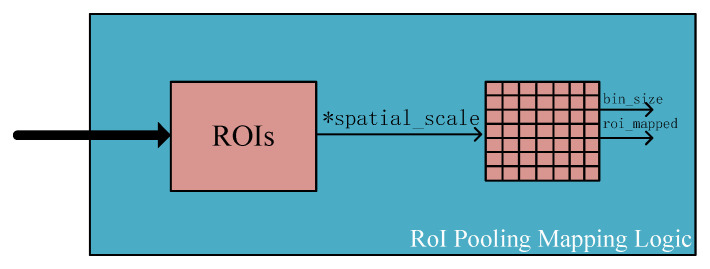
RoI pooling mapping logic.

The third part is the RoI Pooling layer, which uses downsampling of the feature maps of the region of interest generated by the RPN and the shared convolutional layer to further extract the feature maps of the region of interest and send them to the subsequent network. As shown in Figure 2, spatial_scale is the scaling factor of the stride from the first convolutional layer to the shared convolutional layer (the inverse of the product of all strides from the first convolutional layer to the shared convolutional layer). Our goal is to obtain region proposals on the feature map output using the shared convolutional layer, but the size of the region proposals generated by RPN is relative to the original image size. Accordingly, we need to multiply the value of the region proposals by the scaling factor spatial_scale to obtain the mapped coordinates of the region proposals and the size of the sub-grid. As the RoI pooling layer is characterized by more input and less output, we performed a parallel processing on the input and output in the hardware, which can greatly speed up the inference time. This part will be explained in detail in Section 3.2.4.

The classification and regression network is the last part of Faster R-CNN. It uses the candidate regions generated by the RPN to calculate the specific category (such as TV, horse, car, etc.) to which each region proposal belongs through the fc layer and softmax. Bounding box regression is used again to obtain the position offset of each region proposal, which is used to regress a more accurate object detection proposal. However, each region proposal needs to complete the calculation of the fully connected layer, as shown in Table 1. When the number of region proposals is equal to 300, the operations of the Faster R-CNN (vgg16) [4] model are as high as 271.7 G, so our detection accelerator will become very inefficient. Here, we popose the multi-batch RoIs detector method to parallelize and reuse data for different region proposals. This can help networks to increase their speed by up to 11.1×, significantly reducing bandwidth utilization and increasing throughput. This part will be explained in detail in Section 3.3.3.

### 2.2. OpenCL-Based Heterorgeneous Computing Platform Setup

**Figure 3:**
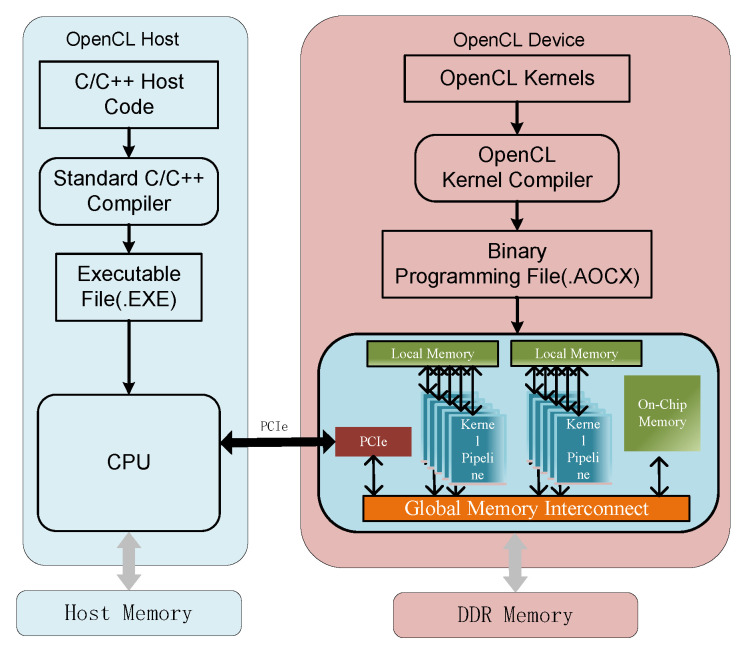
Design flow of OpenCL-based heterogeneous computing platform for Faster R-CNN.

In recent years, with the increasing demand for computing speed and real-time data processing in different fields, the advantages of FPGA’s high degree of parallelism and reconfigurability have gradually emerged. Compared with Register Transfer Level (RTL), such as HDL, High-Level Synthesis (HLS) tools have gradually become dominant in FPGA applications due to their short development cycle and lower research costs. Due to its parallel programming model [27], many people have recently paid more and more attention to the adaptation of the OpenCL heterogeneous computing framework (programming language based on C or C++) in FPGA. As shown in Figure 3, in this article, we used the OpenCL framework to design a Faster R-CNN FPGA accelerator. Generally speaking, this divides the computing system into two parts: the host side and the device side. (a) The host side (usually a CPU processor) is a set of application program interfaces (API) used to define and control the computing platform; (b) the device side (usually FPGA, DSP, GPU, etc.) is used to compile a set of kernel functions to accelerate operations on the FPGA board. The OpenCL device side first sends the data from the DDR memory to the Global Memory, and then communicates with the host side from the Global memory or the local memory through PCIe.

## 3. System Design

### 3.1. Software and Hardware Architecture Co-Design Scheme

**Figure 4:**
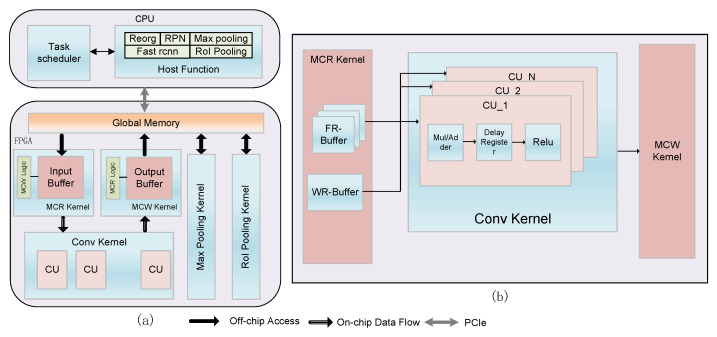
(**a**) Hardware architecture of OpenCL-based Faster R-CNN object detection accelerator. (**b**) The hardware architecture of the convolution kernel.

As shown in Figure 4a, in this article, we propose a software and hardware co-design scheme for Faster R-CNN object detection accelerator. This consists of two parts, the host side and the device side (FPGA). The host side is a series of host task functions running on the CPU, including Reorg function, RPN function, host Max pooling function, Fast R-CNN detection function, host RoI Pooling function, and a task scheduler. The device side is composed of a set of kernel functions with high parallelism running in FPGA, which include Memory Convolution Read (MCR) kernel, Memory Convolution Write (MCW) kernel, Convolution kernel, Max Pooling kernel and RoI Pooling kernel. The proposed software and hardware co-design scheme places the computationally extensive layer on the hardware acceleration device FPGA for execution and places the small computationally complex and logically complex modules(such as RPN, Fast R-CNN detection, etc.) the host side. The specific hardware architecture design and software solutions are described in detail in the following Section 3.2 and Section 3.3.

### 3.2. Hardware Architecture Design

#### 3.2.1. Overall Architecture

As shown in Figure 4a, the proposed Faster R-CNN object detection hardware architecture includes five acceleration kernels, which can implement a series of CNN basic layers, so that we can obtain object detection accelerators for different backbones by adjusting the network configuration parameters. The MCR and MCW kernels are responsible for reading and writing data from the global memory. They are cascaded with the convolution kernel through the OpenCL pipeline, so there is no need to repeatedly transmit the middle-layer feature map data and weight parameters, which will greatly improve the bandwidth utilization of the hardware.

#### 3.2.2. Convolution Kernel

Figure 4b shows the internal structure of the convolution kernel, which reads the vectorized feature map and weight parameters from the input buffer of the MCR kernel through the OpenCL pipeline. We set the degree of parallelism at the Compute Unit(CU) level, which can efficiently accelerate the convolution kernel. Each CU unit is responsible for processing a series of sub-operations, including multiply and accumulate modules, delay registers, and Rectified Linear Unit(Relu) units.

**Figure 5:**
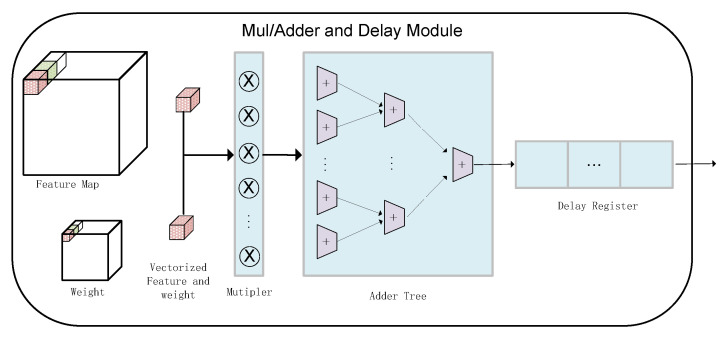
Multiply and accumulate unit of convolutional kernel.

The multiply–accumulate module is shown in Figure 5. The vectorized input feature map and weight parameters are sent to the multiplier and then output to the delay shift register through the addition tree. The reason for designing the delay shift register is that the accumulator will self-add, which will cause the reading and writing of the accumulator results to be in the same memory area, causing memory conflicts. At this time, when a shift register is added after the accumulator, the result of the accumulator forms a pipeline between the accumulator and the shift register, which will greatly improve our convolution kernel execution’s efficiency and throughput.

#### 3.2.3. Max Pooling Kernel

**Figure 6:**
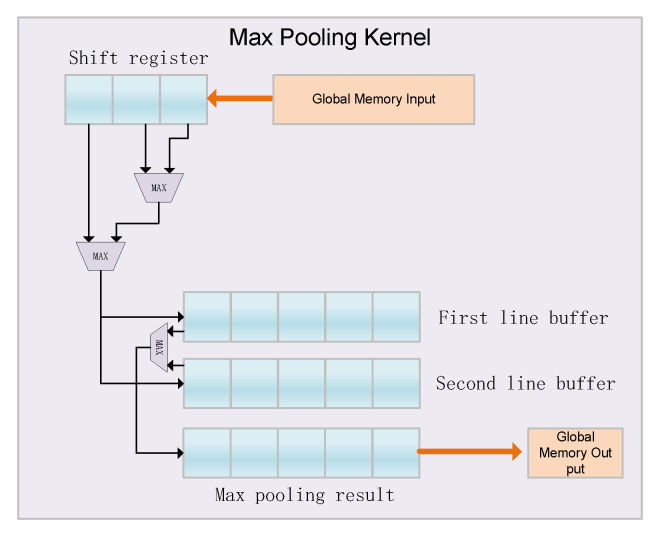
Max Pooling hardware architecture of proposed Faster R-CNN accelerator.

Figure 6 shows the accelerator’s max pooling kernel, which consists of a shift register, a comparator, and two line buffers. The figure shows that the size of the pooling window is 3 × 3. We can see that it first reads data from the global memory and puts them into a shift register of length three. Then the output of the shift register is compared, and the result of the comparison is sent to the two line buffers. Finally, we compare the data in the two row caches again, and the output result is the maximum value of the two row cache data, which is written back to the global memory. From the perspective of the entire architecture, the designed max pooling kernel only delays three clock cycles in the shift register, and will efficiently perform pipeline operations to improve the efficiency of the max pooling kernel.

#### 3.2.4. RoI Pooling Kernel

**Figure 7:**
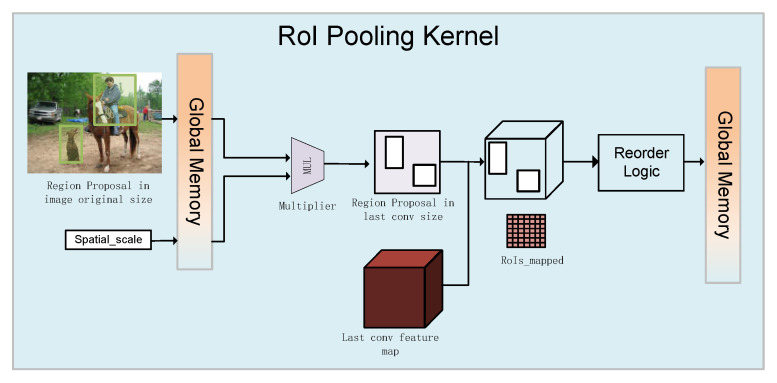
RoI Pooling hardware architecture of proposed Faster R-CNN accelerator.

As shown in Figure 7, we propose a RoI Pooling kernel hardware design based on the NDRange method. First, we used a local work-item to read the region proposal feature map in the global memory and obtain the information of the four coordinates. Then, the region proposals generated by the RPN network (the generated size is based on the size of the original image) were mapped to the size of the last convolution feature map, which was multiplied by the scaling factor spatial_scale. According to the size of the obtained region proposal, the max pooling operation was performed on the feature map of the last convolutional layer. Finally, the output result was reordered and written back to the global memory in the RoI pooling kernel according to the degree of parallelism. In order to improve the concurrent workgroup processing of the kernel, work items were assigned to multiple concurrent workgroups, and the size of each workgroup is (K,K,C). For example, if we process 64 region proposals at a time, then we can map them to a single 3-D dataset with the NDRange size of (8,8,C).

#### 3.2.5. MCR/MCW Kernel

As shown in Figure 4a, the data transfer kernels MCR and MCW are responsible for transferring data between the convolution kernel channel and the global memory. Specifically, MCR transfers the feature map and weight parameters of the image stored in the global memory to the input buffer and then transfers them to the convolution kernel. Similarly, MCW is responsible for writing the feature map data output by the convolution kernel back into the global memory to feed them into the next layer of the network. The data flow on the cache is realized through the OpenCL pipeline, which makes the data flow between the kernels more efficient.

**Figure 8:**
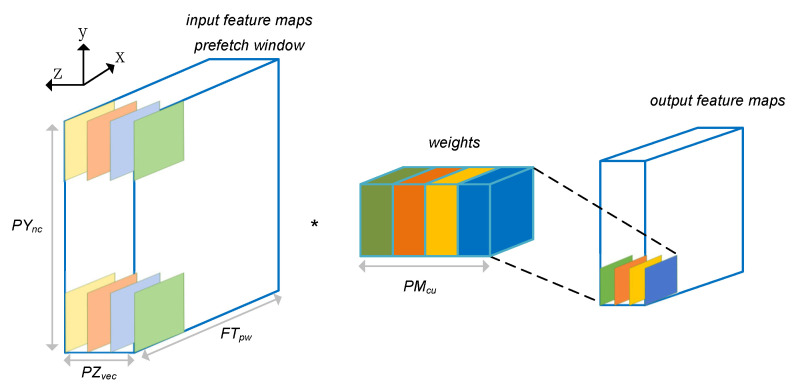
Convolutional weights, input and output feature maps mapping scheme of MCR/MCW Kernel.

For the MCR and MCW cores, we propose a parallel circuit design in the on-chip cache. Figure 8 shows the mapping process of the convolutional layer weights, input and output feature maps in the prefetch window of the MCR/MCW kernel. We design parallelism in three directions, namely, the channel vectorization parallelism $PZ_{vec}$ along the z direction, the parallelism $PY_{nc}$ of multiple convolutions within the prefetch window along the y direction, and parallelism based on the convolution kernel dimension $PM_{cu}$. Specifically, since the convolution is based on the operation of sliding windows, in order to increase the bandwidth utilization, we will read the data of a prefetch window size each time, which vectorizes the data in the z direction; then, multiple convolution operations can be executed in parallel within the prefetch window in the y direction. Finally, in the dimension of the convolution kernel, we perform parallel processing on multiple convolution kernels. For example, we can execute $PM_{cu}$ convolution kernels concurrently.

#### 3.2.6. Buffer Design

**Figure 9:**
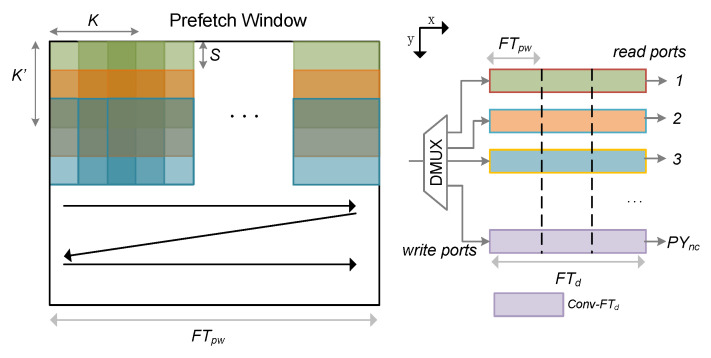
Design and internal structure of the feature map buffer for MCR and MCW kernels. The figure shows the convolution kernel of size $K=K^{{}^{\prime }}=3$, S=1, $PY_{nc}=4$. Each colored box represents the convolution kernels executed in parallel.

In order to achieve simultaneous access and maximum data-sharing of multiple groups of convolutional data on the prefetched window feature map, we propose a single-input and multiple-output line buffer structure to achieve a feature map buffer. As shown in Figure 9, the designed feature map buffer consists of a dual-port RAM, including one write port and multiple read ports. Each time we read feature map data that are equal to the size of the convolution kernel from the prefetch window in Z-order, the sub-window sequentially slides the convolution kernel step size S units along the X axis. In order to avoid memory conflicts caused by repeated readings of the same block address by adjacent convolutions, we read the feature maps of S lines each time and write them into the line buffer in turn. The proposed design can significantly improve the bandwidth utilization of feature map transmission.

### 3.3. Hardware-Aware Algorithm-Level Optimization

#### 3.3.1. Fixed-Point Quantization for Faster R-CNN

Although floating-point numbers can represent higher data accuracy, the implementation of floating-point data on FPGA will use more storage resources and computing resources, which will lead to a longer object detection forward inference time. With the complexity and deepening of the network backbone, the requirements for reasoning delay will become more and more demanding. Therefore, it is extremely important to compress the network. Recent studies [14,28] have shown that using fixed-point formats (8/16bit) instead of floating-point data in FPGAs can significantly reduce bandwidth requirements and dependence on on-chip resources. However, this does not mean that we can use a too-short bit width to represent the weight and activation of the network, because this will cause a serious loss of accuracy. For example, the current binarization research work only uses 1 bit to compress the network model to the extreme, but there will still be a significant decrease in accuracy. At present, there is some research [29,30,31] on ultra-low precision quantization, such as binarization research work, which only uses one bit to compress the network model extreme. However, at present, the gap with the full-precision model is still huge.

In this paper, we extend the dynamic precision data quantification scheme proposed in [14]. Specifically, we performed 8-bit width fixed-point quantization on the weights, input and output of the convolutional layer, as well as the fully connected layer in Faster R-CNN. Since the data of Faster R-CNN on the host side are a floating-point number, we need to convert them to a fixed-point number before they can be sent to the FPGA device. The definition of fixed-point quantization is as follows:

(1) $$Q_{f}={\left(-1\right)}^{s}\cdot 2^{-FL}\sum_{i=0}^{bw-2}2^{i}\cdot B_{i}$$

where $Q_{f}$ denotes the quantized fixed-point number, *s* represents the sign bit, FL indicates that the fractional length may be positive or negative, bw denotes the bit width of the fixed-point number, and $B_{i}$ denotes mantissa.

The goal of quantifying Faster R-CNN object detection is to find the optimal fractional length FL for the model weight parameters, the input and output in each convolution layer or the fully connected layer under the condition of minimal losses of accuracy. They are denoted as $W_{FL}$, $IN_{FL}$, and $OUT_{FL}$, respectively. Specifically, as shown in Algorithm 1, we first set the target bit width bw for the model parameters, the input, and the output of the convolutional layer or the fully connected layer of Faster R-CNN (denoted as $BW_{w}$, $BW_{in}$ and $BW_{out}$), and then traversed this layer by layer until it met the detection accuracy constraints.

Taking model parameters as an example, we set the traversal range to $\left[-R+W_{{\mathrm{FL}}_{\mathrm{init}}}^{i},R+W_{{\mathrm{FL}}_{\mathrm{init}}}^{i}\right]$, where *R* is a threshold, and $W_{{\mathrm{FL}}_{\mathrm{init}}}^{i}$ represents the fractional length initialization of i-layer weights. Here, we set the method of initializing the length of the weight of the *i* layer as follows:

(2) $$W_{{\mathrm{FL}}_{\mathrm{init}}}^{i}={\mathrm{BW}}_{w}-log_{2}\left(max\left(W_{i}\right)+1\right)$$

The input and output settings of the convolutional layer and the fully connected layer were the same as the parameters. Here, we set the traversal range to be very small (usually set to 3). This will not affect the accuracy losses of Faster R-CNN, which will greatly improve the efficiency of our experiment. Table 2 shows the quantization results of the Faster R-CNN object detection framework based on different network backbones. The model with the result of vgg16 was used as the backbone network, and compressed four times, from the original 137.1 MB to 34.3 MB. Therefore, fixed-point quantization can compress the model to accelerate the Faster R-CNN object detection accelerator.
**Algorithm 1:** Fixed-Point Quantization Algorithm Flow For Faster R-CNN
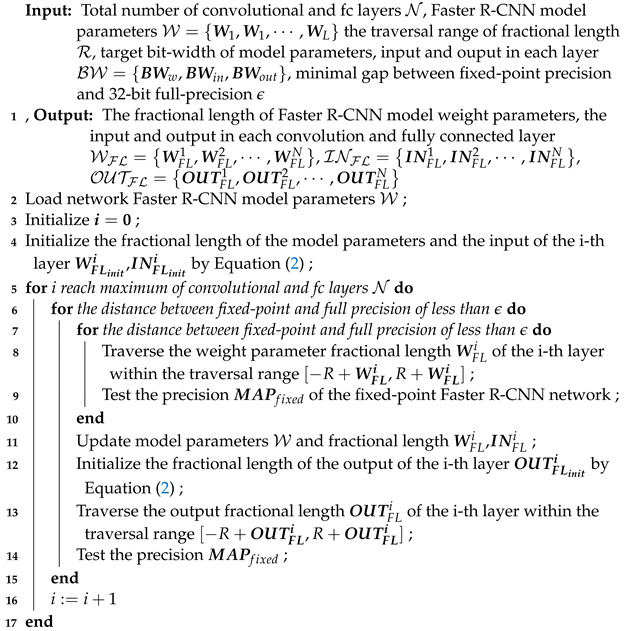


#### 3.3.2. Layer Fusion

The convolution layer convolves the input feature map with the convolution kernel to obtain the output feature map. This is actually a three-dimensional multiply–accumulate operation, defined as follows:

(3) $$f_{n,i,j}^{out}=\sum_{c=0}^{C-1}\sum_{k_{x}=0}^{K-1}\sum_{k_{y}=0}^{K-1}f_{c,i\cdot S+k_{x},j\cdot S+k_{y}}^{in}\times W_{n,c,k_{x},k_{y}}$$

where $f_{c,i\cdot S+k_{x}j\cdot S+k_{y}}^{in}$ and $f_{n,i,j}^{out}$ represent the input and the output feature map of the convolutional layer, respectively. $W_{n,c,k_{x},k_{y}}$ denotes model parameters.

The Batch Normalization (BN) layer is used by many deep models due to its ability to speed up convergence and prevent gradient explosion or gradient disappearance. Generally speaking, the BN layer is behind the convolutional layer, which allows us to directly embed the operations of the BN layer into the convolutional layer during the inference stage of the network. This can effectively reduce the amount of network calculations, and can also increase the network’s reasoning time. Specifically, in the inference stage, the definition of the BN layer is as follows:

(4) $$f^{BN}=\gamma \times \frac{f^{in}-\mu }{\sqrt{\sigma^{2}+\epsilon }}+\beta$$

where $f^{in}$ is input of the BN layer (the output of the convolutional layer), $f^{BN}$ represents the output of the BN layer, and μ represents the average and variance of the mini-batch. γ is the scaling factor, β is the translation factor, and ϵ avoids the minimum value set by the division by zero.

We further expand the above Equation (4):

(5) $$f^{BN}=\frac{\gamma }{\sqrt{\sigma^{2}+\epsilon }}\times f^{in}+\beta -\frac{\mu }{\sqrt{\sigma^{2}+\epsilon }}$$

Therefore, we can transform this into the following:

(6) $$f^{BN}=W^{BN}\times f^{in}+b$$

where

(7) $$W^{BN}=\frac{\gamma }{\sqrt{\sigma^{2}+\epsilon }},b=\beta -\frac{\mu }{\sqrt{\sigma^{2}+\epsilon }}$$

Then, the weight of the fusion of the BN layer and the convolutional layer is a simple multiplication of the two-layer parameter:

(8) $$W^{fusion}=W_{n,c,k_{x},k_{y}}\cdot W^{BN}$$

The experimental results show that the layer fusion optimization has no accuracy loss, but provides great benefits in the utilization of hardware resources.

#### 3.3.3. Multi-Batch RoIs Detector

**Figure 10:**
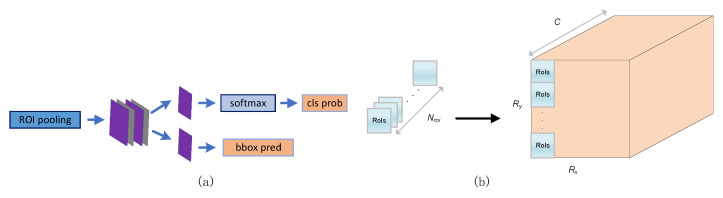
(**a**) Classification and regression network. (**b**) Based on hardware-aware Multi-batch RoIs’ detector algorithm optimization.

From Figure 10a, we know that the region proposals (denoted as Rois) obtained through the RoI pooling layer are used in the subsequent detection phase (including two fully connected layers and two 1 × 1 convolutional layers). As shown in Figure 10b, since Rois is an independent execution detection phase, we propose a multi-batch RoIs detector method for this feature in this article. Specifically, assuming that the total of $N_{rois}$ region proposals are output and $N_{rois}$ can perform square root rounding operations, we can rearrange these into the frame on the right side of Figure 10b, where $N_{rois}=R_{x}\times R_{y}$. Through this reordering, we can transform the original serial execution of multiple region proposals into a stage outputting multiple region proposals at once. This can help the network to achieve a speedup gain of up to 11.1×, which greatly reduces bandwidth utilization and increases throughput. Section 5.2 demonstrates the effectiveness of the approach.

## 4. Performance Modeling

Maximizing the performance of the designed Faster R-CNN object detection accelerator while being constrained by the limited resources of the FPGA is a formidable challenge. Synthesis fails because FPGA synthesis runs for a long time (maybe hours) or because of insufficient hardware resources. Compiling for every combination of hardware parameters is unwise and unfeasible. Therefore, this paper models performance and bandwidth for rapid design space exploration. We assume that the input feature map size of the *l* layer of our network is $N_{l}\times P_{l}\times C_{l}$, the size of the convolution kernel is $K_{l}\times K_{l}^{{}^{\prime }}\times C_{l}$, the step size is $S_{l}$, and the output feature map size is $N_{l}^{{}^{\prime }}\times P_{l}^{{}^{\prime }}\times C_{l}^{{}^{\prime }}$.

Figure 8 shows that the designed accelerator has three dimensions of parallelism, which are based on the parallelism of different convolution kernels ($PM_{cu}$), the parallelism $PY_{nc}$ of multiple convolutions within the prefetch window along the y direction and the channel vectorization parallelism $PZ_{vec}$ along the z direction. Choosing the best combination of design variables ($PM_{cu}$, $PY_{nc}$, $PM_{cu}$) can maximize the performance of the Faster R-CNN accelerator. Since the fully connected layer can be regarded as a convolutional layer of 1×1, we model the running time of the convolutional or fully connected layer under the condition of FPGA resource constraints:

(9) $$\begin{array}{c} R_{time}^{l}=\frac{\#\;\mathrm{Operations}{\;}^{l}}{PM_{cu}\;\times \;PZ_{vec}\;\times \;PY_{nc}\;\times \;Clock}\\ \min_{PM_{cu},PZ_{vec},PY_{nc}}\sum_{l=1}^{L}\left(R_{time}^{l}\right)\\ \;s.t.\;R_{use}\leq MAX_{RC} \end{array}$$

where $\#Operations^{l}=N_{l}^{{}^{\prime }}\times P_{l}^{{}^{\prime }}\times C_{l}^{{}^{\prime }}\times K_{l}\times K_{l}^{{}^{\prime }}\times C_{l}$ represents the *l* layer operations, Clock indicates the clock frequency at which the accelerator works. $R_{use}$ represents the number of FPGA resources consumed by the designed accelerator to run, including DSP, logic resources, and on-chip memory, and $MAX_{RC}$ represents the total number of resources that are actually owned by a given FPGA. The total time of the other functional layers is insignificant when compared to the total runtime, so the total throughput can be evaluated as:

(10) $$THP_{\mathrm{total}\;}=\frac{1}{\sum_{l=1}^{L}\left(R_{\mathrm{time}}^{l}\right)}$$

The detailed design space exploration process and resource exploration are elaborated in the following experimental Section 5.2, and we compare the obtained theoretical time with the time measured on the board on an actual FPGA.

## 5. Results

### 5.1. Experimental Setup

To evaluate the performance of the proposed object detection accelerator, we implemented the design on Intel Arria-10 GX FPGA Development Kit. The FPGA device has 427 K Adaptive Logic Modules, 1518 DSP blocks, 66 Mb of on-chip memory, 2 GB of off-chip memory, and external memory DDR. The bandwidth is 19.2 GB/s. The FPGA board is equipped with an Intel i9-9900k CPU and 64 GB memory on the workstation. The proposed framework adopts a high-level synthesis (HLS)-based design method, and its OpenCL kernel code is compiled on Intel FPGA OpenCL SDK v20.1. The host-side CPU executes the host program and the device-side FPGA executes the kernel code with a large amount of computation, such as convolutional layers, fully connected layers, etc. We selected two backbone networks (resnet50 [3] and vgg16 [2]) to test the performance of the Faster R-CNN accelerator. The Pascal VOC2007 dataset [32] was used to measure the detection accuracy of Faster R-CNN. We quantized the Faster R-CNN model with 8-bit precision, and the accuracy dropped by less than 1%.

### 5.2. Design Space Exploration

**Figure 11:**
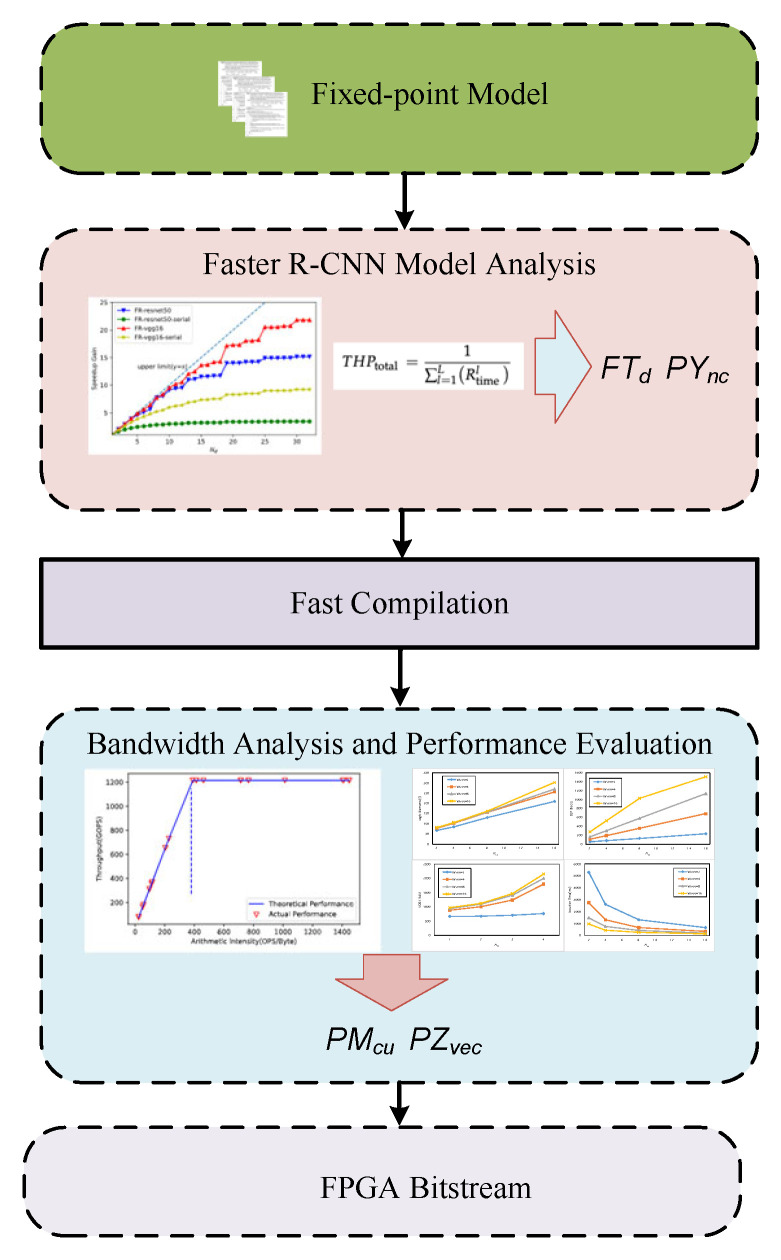
Design space exploration flow.

We developed an automatic design space exploration engine based on a python script to fully and reasonably use hardware resources. As shown in Figure 11, this solution can automatically load the fixed-point model to quickly compile multiple accelerator kernel codes. Then, we analyzed and counted the consumed hardware resources using compilation report and selected the best theoretical performance that met the expectations to execute the complete compilation and synthesis process, and finally generate the FPGA bitstream file.

**Figure 12:**
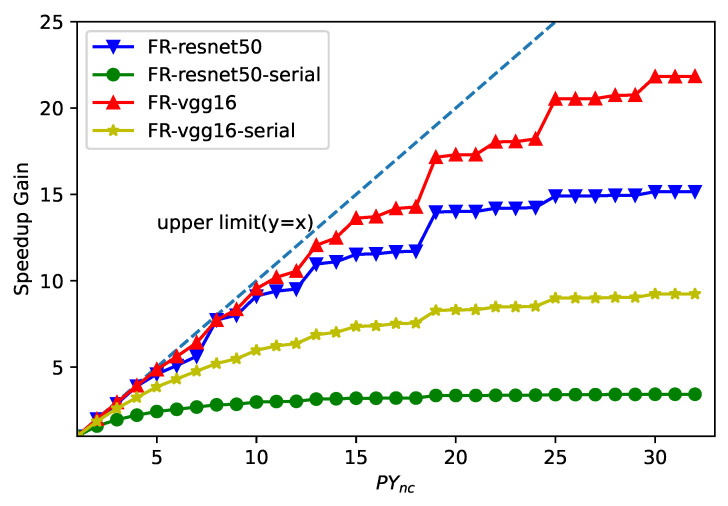
Exploration of the optimal value of $PY_{nc}$ under the frequency of 200 MHz.

Specifically, we first analyzed the Faster R-CNN model from the perspective of performance modeling. For a specific network later, the speedup gain introduced by the parallel data flow along the y direction iwa affected by the ratio $N^{{}^{\prime }}/PY_{nc}$. Figure 12 shows the speedup gain curves of the Faster R-CNN model under different degrees of parallelism $PY_{nc}$. We can see that the speedup gain of different backbone network models rapidly decreased when $PY_{nc}>14$. For large values of $PY_{nc}$, the increase in speed drops rapidly for layers with small feature maps along the y direction. Therefore, we chose $PY_{nc}=14$ as the best configuration for hardware parameters. As shown in Figure 9, the line buffer of the feature map should accommodate the prefetch window of one line, and the buffer depth $FT_{d}$ of the feature map should satisfy $FT_{d}\geq FT_{pw}\cdot S\cdot C$. In this way, we can obtain the optimal $FT_{d}$ in the model. From Figure 12, we can also see that the proposed multi-batch RoIs’ detector method can greatly improve the speed. For example, when $PY_{nc}=14$, the speed using this method increased by 11.1× (blue line in the figure), while the speed when not using this method only increased by 3.2× (green line in Figure 12).

**Figure 13:**
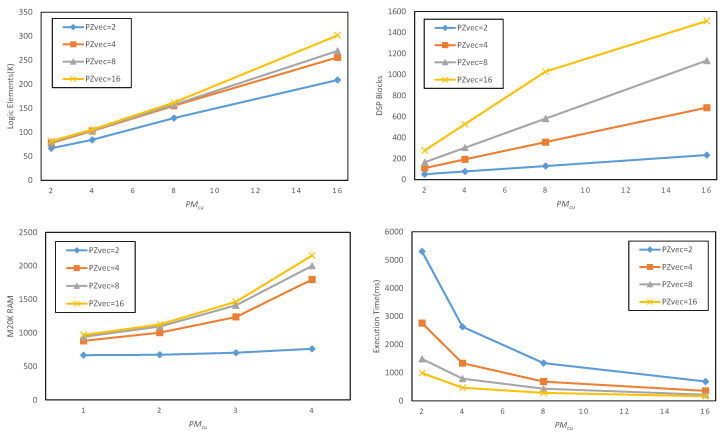
Design space exploration results for proposed Faster R-CNN object detection accelerator using resnet50 [3] model with $PY_{nc}=14$ and the frequency is 200 MHz.

Then, we quickly compiled the kernel code for the target FPGA device multiple times, and obtained the consumed hardware resource information such as DSP, on-chip storage, and logic from the compilation report. The huge advantage of this fast compilation is that the model can be generated quickly through the python scripting language. Figure 13 shows the design space exploration results of the proposed Faster R-CNN accelerator, and the average execution time per image is calculated by Equation (9). We can see that when $PM_{cu}=16,PZ_{vec}=16$, the DSP utilization of the target device Arria-10 GX1150 FPGA exceeds 99%; therefore, when we increase the parallelism, the compilation will fail. Therefore, for the Faster R-CNN network whose backbone network is resnet50, the hardware parameters are configured as $PM_{cu}=16,PZ_{vec}=16$ to maximize the performance and resource utilization of the accelerator.

### 5.3. Comparison with Estimated Performance

**Figure 14:**
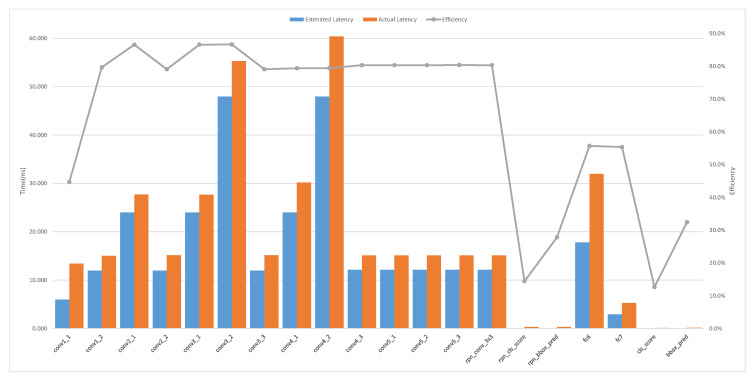
Comparison of the efficiency of the convolution computation for each layer of the Faster R-CNN and the backbone network is vgg16 [2]. The estimated time is calculated using the theoretical performance model, and the actual time is on the Faster R-CNN-vgg16 design.The hardware configure parameters is $PY_{nc}=14,PM_{cu}=16,PZ_{vec}=8$.

As shown in Figure 14, we obtained the theoretical execution time of convolutional and fully connected layers from Equation (9) for performance modeling, and compared this with the actual execution time in the designed accelerator. From Figure 14, we can observe that the execution efficiency of most convolutional layers is about 80%. The main reason for this is that the size of the convolution kernel is 1×1, which means that the calculation amount on the FPGA chip is too small. In this case, the accelerator transmits data most of the time; that is to say, the computing unit on the FPGA chip is waiting most of the time instead of working, resulting in a low utilization of the accelerator’s core channel pipeline.

### 5.4. Comparison with Start-of-the-Art

As shown in Table 3, we first compared the proposed accelerator design with the state-of-the-art Faster R-CNN accelerator with the same backbone network on different acceleration platforms. Using the premise that the backbone networks are all vgg16 [2], we achieved the highest detection accuracy, while the detection speed was 3.5× of work [16] and 2.7× of work [18]. On this basis, we achieved a full throughput improvement of 10× over work [18]. Second, we compared the YOLO family of state-of-the-art one-stage detectors, whose designs employ different types of convolution strategies, including spatial convolution [19], frequency domain convolution [24], and multiplication-free binary convolution [17]. As shown in Table 1, since the input image resolution of Faster R-CNN is larger than that of the one-stage detector, the computation of Faster R-CNN ranges from about 3 to 6 times larger than that of YOLO. Therefore, even though our designed Faster R-CNN accelerator has a lower detection speed than the YOLO accelerator, the proposed design comparison work [17] achieves a performance improvement of 2.1× in terms of throughput.

Finally, the first row of Table 4 shows a comparison with the results achieved on NVIDIA K40 GPU [4]. When the same backbone network is run vgg16 [2], the results show that our designed Faster R-CNN accelerator achieved a 5.7× improvement in power efficiency at the cost of a 1-point drop in detection accuracy. Compared with GPU, the hardware accelerator designed in this paper, which can be deployed on FPGA, has more flexibility and a higher practical application value.

## 6. Conclusions

This work proposes a high-throughput Faster R-CNN object detection accelerator design. The hardware architecture designed in this paper adopts a series of flexible and scalable kernel pipeline designs to support Faster R-CNN architectures of different backbone networks such as resnet50, vgg16, etc. Through an 8 bit width quantization, layer fusion, and, ulti-batch RoIs detector method, the resource utilization of the hardware is greatly improved. We also propose end-to-end design space exploration, and the experimental results show that our design achieves a 10x improvement in inference throughput compared to state-of-the-art designs.

For future studies, we intend to sparse the Faster R-CNN design and achieve a higher compression ratio, using the pruning algorithm to achieve a more efficient Faster R-CNN detection accelerator.

## Figures and Tables

**Table 1:** Statistics of operation, parameters and mean average precision(mAP) accuracy of different object-detection models.

Model	Number of Region Proposal	Resolution	# Param	# Operation	mAP
Yolo [6]	-	448 × 448	146.2 MB	40.3 G	63.4%
Yolov2 [25]	-	4808 × 480	50.6 MB	39.1 G	77.8%
Yolov2 Tiny [25]	-	4168 × 416	27.1 MB	29.4 G	57.2%
Faster-R-CNN(vgg16) [4]	300	8008 × 600	137.1 MB	271.7 G	69.9%
Faster-R-CNN(resnet50) [4]	300	8008 × 600	236 MB	215.6 G	73.7%

**Table 2:** Faster R-CNN 8-bit quantization result.

Model	float32 mAP	FP8 mAP	# Param	# Param Quantized
Faster-RCNN-vgg16	69.0%	68.3%	137.1 MB	34.3 MB
Faster-RCNN-resnet50	73.7%	73.4%	236 MB	59 MB

**Table 3:** Comparison with the state-of-the-art object detection FPGA accelerators.

Implementation	Resolution	Precision	Target Device	Logic Usage	DSP Usage	RAM Usage	Clock	Latency (ms)	Throughput (GOP/s)	Accuracy (mAP)	Power(W)
Faster R-CNN (vgg16) [16]	-	32(float)	Xilinx ZC706	-	-	-	200 MHz	875	-	-	1.167
Faster R-CNN [18]	-	8(fixed)	Zynq 7045	-	-	-	-	680	86.8	66	3
YOLOv1 [18]	416 × 416	8(fixed)	Xilinx KU115	-	-	-	-	65	461.5	62	13
Lightweight YOLOv2 [17]	224 × 224	1-16(fixed)	Zynq MPSoC	-	-	-	300 MHz	24.5	408.1	67.6	4.5
OpenCL-YOLOv2 [19]	416 × 416	8(fixed)	Arria-10 GX1150	34%	72%	68%	200 MHz	53	566	76	26
FCLCNN [24]	416 × 416	16(fixed)	Arria-10 GX1150	50%	98%	88%	278 MHz	53.8	557.6	76.08	45
Proposed Faster R-CNN(resnet50)	800 × 600	8(fixed)	Arria-10 GX1150	71%	99%	72%	172 MHz	153.6	719	73.4	26
Proposed Faster R-CNN(vgg16)	800 × 600	8(fixed)	Arria-10 GX1150	71%	99%	72%	172 MHz	248.7	864.9	68.3	26

**Table 4:** Comparison with the baseline GPU imlpementation.

Implementation	Resolution	Precision	Target Device	Latency (ms)	Throughput (GOP/s)	Accuracy (mAP)	Power Efficiency (GOP/s/W)
Faster R-CNN GPU basline [4]	800 × 600	32(float)	Nvidia K40	198	1372.2	69.9	5.83
Proposed Faster R-CNN(resnet50)	800 × 600	8(fixed)	Arria-10 GX1150	153.6	719	73.4	27.7
Proposed Faster R-CNN(vgg16)	800 × 600	8(fixed)	Arria-10 GX1150	248.7	864.9	68.3	33.3

## Data Availability

Not applicable.

## References

[1] Krizhevsky A., Sutskever I., Hinton G.E. (2012). Imagenet classification with deep convolutional neural networks. Adv. Neural Inf. Process. Syst..

[2] Simonyan K., Zisserman A. (2014). Very deep convolutional networks for large-scale image recognition. arXiv.

[3] He K., Zhang X., Ren S., Sun J. Deep residual learning for image recognition. Proceedings of the IEEE Conference on cOmputer Vision and Pattern Recognition.

[4] Ren S., He K., Girshick R., Sun J. (2015). Faster r-cnn: Towards real-time object detection with region proposal networks. Adv. Neural Inf. Process. Syst..

[5] Girshick R. Fast r-cnn. Proceedings of the IEEE International Conference on Computer Vision.

[6] Redmon J., Divvala S., Girshick R., Farhadi A. You only look once: Unified, real-time object detection. Proceedings of the IEEE Conference on Computer Vision and Pattern Recognition.

[7] Girshick R., Donahue J., Darrell T., Malik J. Rich feature hierarchies for accurate object detection and semantic segmentation. Proceedings of the IEEE Conference on Computer Vision and pAttern Recognition.

[8] Abdel-Hamid O., Mohamed A.R., Jiang H., Deng L., Penn G., Yu D. (2014). Convolutional neural networks for speech recognition. IEEE/ACM Trans. Audio Speech Lang. Process..

[9] Guo K., Zeng S., Yu J., Wang Y., Yang H. (2019). [DL] A survey of FPGA-based neural network inference accelerators. ACM Trans. Reconfigurable Technol. Syst. (TRETS).

[10] Zhang C., Li P., Sun G., Guan Y., Xiao B., Cong J. Optimizing fpga-based accelerator design for deep convolutional neural networks. Proceedings of the 2015 ACM/SIGDA International Symposium on fIeld-Programmable Gate Arrays.

[11] Wang D., An J., Xu K. (2016). PipeCNN: An OpenCL-based FPGA accelerator for large-scale convolution neuron networks. arXiv.

[12] Wang D., Xu K., Jia Q., Ghiasi S. ABM-SpConv: A Novel Approach to FPGA-Based Acceleration of ConvolutionaI NeuraI Network Inference. Proceedings of the 2019 56th ACM/IEEE Design Automation Conference (DAC).

[13] Suda N., Chandra V., Dasika G., Mohanty A., Ma Y., Vrudhula S., Seo J.S., Cao Y. Throughput-optimized OpenCL-based FPGA accelerator for large-scale convolutional neural networks. Proceedings of the 2016 ACM/SIGDA International Symposium on Field-Programmable Gate Arrays.

[14] Qiu J., Wang J., Yao S., Guo K., Li B., Zhou E., Yu J., Tang T., Xu N., Song S. Going deeper with embedded fpga platform for convolutional neural network. Proceedings of the 2016 ACM/SIGDA International Symposium on Field-Programmable Gate Arrays.

[15] Zeng H., Chen R., Zhang C., Prasanna V. A framework for generating high throughput CNN implementations on FPGAs. Proceedings of the 2018 ACM/SIGDA International Symposium on Field-Programmable Gate Arrays.

[16] Zhao R., Niu X., Wu Y., Luk W., Liu Q. Optimizing CNN-based object detection algorithms on embedded FPGA platforms. Proceedings of the International Symposium on Applied Reconfigurable Computing. Springer.

[17] Nakahara H., Yonekawa H., Fujii T., Sato S. A lightweight YOLOv2: A binarized CNN with a parallel support vector regression for an FPGA. Proceedings of the 2018 ACM/SIGDA International Symposium on Field-Programmable Gate Arrays.

[18] Yu J., Guo K., Hu Y., Ning X., Qiu J., Mao H., Yao S., Tang T., Li B., Wang Y. Real-time object detection towards high power efficiency. Proceedings of the 2018 Design, Automation & Test in Europe Conference & Exhibition (DATE).

[19] Xu K., Wang X., Liu X., Cao C., Li H., Peng H., Wang D. (2021). A dedicated hardware accelerator for real-time acceleration of YOLOv2. J. -Real-Time Image Process..

[20] Ding C., Wang S., Liu N., Xu K., Wang Y., Liang Y. REQ-YOLO: A resource-aware, efficient quantization framework for object detection on FPGAs. Proceedings of the 2019 ACM/SIGDA International Symposium on Field-Programmable Gate Arrays.

[21] Wang Z., Xu K., Wu S., Liu L., Liu L., Wang D. (2020). Sparse-YOLO: Hardware/Software co-design of an FPGA accelerator for YOLOv2. IEEE Access.

[22] Yu L., Zhu J., Zhao Q., Wang Z. (2022). An Efficient YOLO Algorithm with an Attention Mechanism for Vision-Based Defect Inspection Deployed on FPGA. Micromachines.

[23] Pestana D., Miranda P.R., Lopes J.D., Duarte R.P., Véstias M.P., Neto H.C., De Sousa J.T. (2021). A full featured configurable accelerator for object detection with YOLO. IEEE Access.

[24] Xu X., Liu B. FCLNN: A flexible framework for fast CNN prototyping on FPGA with OpenCL and caffe. Proceedings of the 2018 International Conference on Field-Programmable Technology (FPT).

[25] Redmon J., Farhadi A. YOLO9000: Better, faster, stronger. Proceedings of the IEEE Conference on Computer Vision and Pattern Recognition.

[26] Uijlings J.R., Van De Sande K.E., Gevers T., Smeulders A.W. (2013). Selective search for object recognition. Int. J. Comput. Vis..

[27] Khronos OpenCL Working Group (2011). The OpenCL Specification Version 1.1. http://www.khronos.org/registry/cl/specs/opencl-1.1.pdf.

[28] Gysel P., Pimentel J., Motamedi M., Ghiasi S. (2018). Ristretto: A framework for empirical study of resource-efficient inference in convolutional neural networks. IEEE Trans. Neural Networks Learn. Syst..

[29] Rastegari M., Ordonez V., Redmon J., Farhadi A. (2016). Xnor-net: Imagenet classification using binary convolutional neural networks. Proceedings of the European Conference on Computer Vision.

[30] Liu Z., Wu B., Luo W., Yang X., Liu W., Cheng K.T. Bi-real net: Enhancing the performance of 1-bit cnns with improved representational capability and advanced training algorithm. Proceedings of the European Conference on Computer Vision (ECCV).

[31] Leng C., Dou Z., Li H., Zhu S., Jin R. Extremely low bit neural network: Squeeze the last bit out with admm. Proceedings of the Thirty-Second AAAI Conference on Artificial Intelligence.

[32] Everingham M., Van Gool L., Williams C.K., Winn J., Zisserman A. (2010). The pascal visual object classes (voc) challenge. Int. J. Comput. Vis..

